# Mechanisms of Macrophage Polarization in Insulin Signaling and Sensitivity

**DOI:** 10.3389/fendo.2020.00062

**Published:** 2020-02-19

**Authors:** Lucie Orliaguet, Elise Dalmas, Karima Drareni, Nicolas Venteclef, Fawaz Alzaid

**Affiliations:** ^1^Centre de Recherche des Cordeliers, INSERM, Sorbonne Université, USPC, Université Paris Descartes, Université Paris Diderot, Paris, France; ^2^Institute for Diabetes, Obesity and Metabolism, University of Pennsylvania, Philadelphia, PA, United States

**Keywords:** macrophage, inflammation, type-2 diabetes, adipose tissue, liver, pancreas, immunometabolism

## Abstract

Type-2 diabetes (T2D) is a disease of two etiologies: metabolic and inflammatory. At the cross-section of these etiologies lays the phenomenon of metabolic inflammation. Whilst metabolic inflammation is characterized as systemic, a common starting point is the tissue-resident macrophage, who's successful physiological or aberrant pathological adaptation to its microenvironment determines disease course and severity. This review will highlight the key mechanisms in macrophage polarization, inflammatory and non-inflammatory signaling that dictates the development and progression of insulin resistance and T2D. We first describe the known homeostatic functions of tissue macrophages in insulin secreting and major insulin sensitive tissues. Importantly we highlight the known mechanisms of aberrant macrophage activation in these tissues and the ways in which this leads to impairment of insulin sensitivity/secretion and the development of T2D. We next describe the cellular mechanisms that are known to dictate macrophage polarization. We review recent progress in macrophage bio-energetics, an emerging field of research that places cellular metabolism at the center of immune-effector function. Importantly, following the advent of the metabolically-activated macrophage, we cover the known transcriptional and epigenetic factors that canonically and non-canonically dictate macrophage differentiation and inflammatory polarization. In closing perspectives, we discuss emerging research themes and highlight novel non-inflammatory or non-immune roles that tissue macrophages have in maintaining microenvironmental and systemic homeostasis.

## Introduction: Inflammation in Insulin Secretion, Sensitivity and Resistance

**Figure 1:**
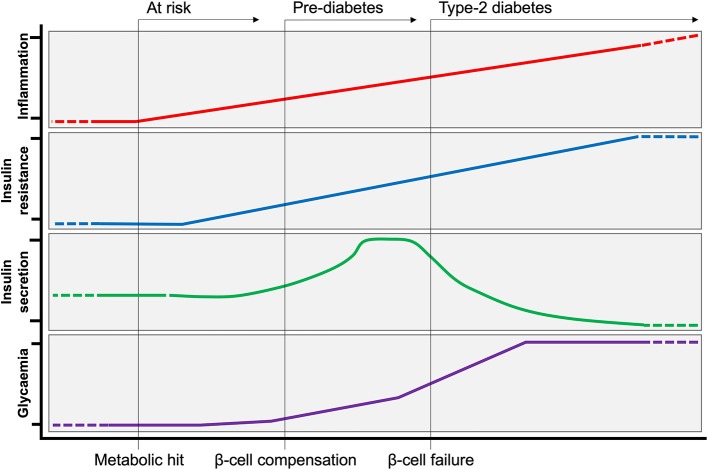
Evolution of Type-2 Diabetes. Following a metabolic hit, inflammation is at the initial steps of developing type-2 diabetes (T2D). Peripheral insulin resistance develops in tandem with increasing inflammation. Insulin resistance is initially met by a compensatory response from the pancreas, producing more insulin to maintain normoglycemia (pre-diabetes). Over time, insulin producing β-cells can no longer cope with increased demand, and insulin production ceases (β-cell failure). At this stage of persistent hyperglycaemia T2D is established.

Type-2 diabetes (T2D) is a disease with dual etiologies, inflammatory, and metabolic. Over the past 20 years, inflammation has gained increasing recognition for the important role it plays in increasing risk of insulin resistance and can be seen as an aetiological starting point for metabolic decline. Several studies have attempted to define the kinetics between inflammation and insulin resistance, where some report local insulin resistance preceding inflammation (1) and others reporting inflammation prior to insulin resistance (2). However, blunting inflammatory responses has consistently been reported as metabolically protective, mitigating the development of insulin resistance and T2D. Thus, inflammation is seen decisive factor in losing tolerance to metabolic dysregulation. Insulin resistance in the liver, adipose tissue and skeletal muscle is initially met with a burst of activity from the pancreas that maintains normal levels of glycaemia (the pre-diabetic stage) (3–5). When this stage is prolonged and insulin production can no longer meet demands, frank T2D develops and predisposes individuals to a variety of complications and comorbidities (Figure 1). These complications and comorbidities are broadly hepatic and cardiovascular in nature and are directly related to increasing inflammation, hyperglycaemia, and dyslipidemia. The following review addresses the various mechanisms and roles of inflammation in the development of T2D with a particular focus on the liver, adipose tissue and the pancreas.

### Inflammation and Metabolic Health

The first evidence linking inflammation to metabolic health dates back to 1993 when Gokhan Hotamisligil and Bruce Spiegelman discovered the increasing expression of pro-inflammatory cytokine tumor necrosis factor (TNF)-α in adipose tissue (AT) of rodent models of obesity (6). Neutralizing TNF-α in obese rats led to a significant increase in glucose uptake in response to insulin. Their study showed that blocking a single cytokine can restore insulin sensitivity. A decade later, macrophages were identified as the main source of TNF-α and other pro-inflammatory molecules (IL-6 and iNOS) in obesity (7). Moreover, macrophages drastically accumulate in adipose tissue during obesity and at the onset of insulin resistance. These early studies brought-to-light the contribution of inflammation to metabolic decline associated with insulin resistance and T2D.

Since these findings, the immune system has gained considerable attention as a major regulator of metabolic homeostasis. Innate immune cells, namely macrophages, reside in all the metabolic tissues that coordinate glycemic homeostasis, namely AT, liver, and pancreas. Tissue-resident innate immune cells form a *bona fide* tissue-specific immune niche, with each niche having its particularities to cope with microenvironmental cues. Macrophages are by far the most studied and proportionally numerous innate immune cell type [25% of AT innate immune cells (8), 20–35% of the non-parenchymal hepatic cells in the liver (9), up to 90% of immune cells in pancreatic islets (10)].

### Macrophage Polarization: Regulation of Acute and Chronic Inflammation

Macrophages were firstly identified by Ellie Metchnikoff as phagocytic cells. They form part of the myeloid lineage and are capable of rapidly mounting non-specific responses to a wide range of pathogens. Phagocytosis is a cellular process associated with innate immune responses to pathogens, is critical in the clearance of cellular debris, tissue repair, and maintaining tissue homeostasis throughout the organism. Tissue-resident macrophages develop from progenitors in the yolk sac, fetal liver, and from circulating monocytes that originate in bone marrow (11). Under physiological conditions, tissue-resident macrophages play a key role in the maintenance of the integrity and homeostasis of their respective tissues.

Macrophages quickly respond to environmental cues and consequently adapt their function, they sense changes in their microenvironment through cell surface receptor engagement. The main receptors relaying environmental signals are toll-like receptors (TLRs), which form part of the larger family of pattern recognition receptors (PRRs). Ligation of TLRs/PRRs by damage- or pathogen-associated molecular patterns (DAMP/PAMPs) present in the microenvironment activates transcriptional programs in macrophages to mount an adapted functional phenotype (12). Whilst these transcriptional mechanisms have been well-described (and are addressed in this review), macrophages also extensively adapt their cellular metabolism to meet the bioenergetic needs and optimize effector function (13). The latter has gained much attention in recent research.

**Figure 2:**
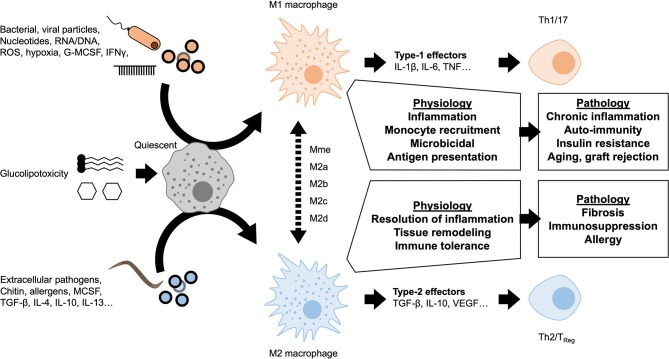
Macrophage polarization and chronic inflammation. A variety of stimuli are known to induce either M1-like pro-inflammatory or M2-like anti-inflammatory polarization. These polarization states represent the extremes of a spectrum of activation profiles, dependent on nature of the stimulus and microenvironmental factors. Self-resolving inflammation is transient and considered a necessary physiological response in maintaining homeostasis and host-defense. If polarization is persistent, downstream signaling from macrophages also leads to lymphocyte polarization. Sustained dysregulated macrophage activation and lymphocytic polarization are important parts of a number of pathologies.

A dichotomy is currently used to describe macrophage polarization states: M1 as pro-inflammatory or classically activated vs. M2 as anti-inflammatory or alternatively activated (Figure 2). The nomenclature of these subsets derives from the type-1 or type-2 immune responses canonically associated with signaling molecules released upon polarization. Macrophage signaling also polarizes the adaptive immune compartment to maintain a chronic T helper (Th)1/17 or Th2 response. The M1 polarization state is associated with a type-1 response (Th1/17) and the production of pro-inflammatory mediators associated with bacterial or viral responses. M1 macrophages have strong microbicidal and antigen presenting capacities. They produce powerful pro-inflammatory cytokines such as TNF-α, IL-6, IL-1β, and reactive oxygen species (ROS). M2 macrophages elicit type-2 signaling, typically in response to extracellular pathogens (helminths, parasites), producing anti-inflammatory mediators such as IL-10 and TGF-β. M2 polarization is also considered a pro-resolution response, associated with later stages of resolving inflammation. The adaptive immune system appropriately undergoes Th2 polarization producing regulatory and remodeling cytokines such as IL-4, IL-5, and IL-13. Accordingly, the immunoregulatory response has been attributed to the specialized regulatory T-cells (T_Reg_) subpopulation. The pro-resolution response can manifest as scarring or tissue remodeling, which when aberrant causes tissue fibrosis, type-2 effector molecules also exacerbate allergic responses (14). Whilst the discrete M1 and M2 classification remains in use today, underlying this dichotomy exists a continuum of diverse responses and intermediate macrophage phenotypes. Novel functional classifications represent polarized macrophages along a sliding scale between M1 and M2 depending on chemokine/cytokine secretion, transcription factor engagement and more recently on the cellular metabolic phenotype (15). The rise of single-cell sequencing and of mass cytometry (CyTOF) are coming a long way to deciphering the functional diversity and plasticity of macrophages (16).

## Tissue Macrophages in Metabolic Physiology and Physiopathology

**Figure 3:**
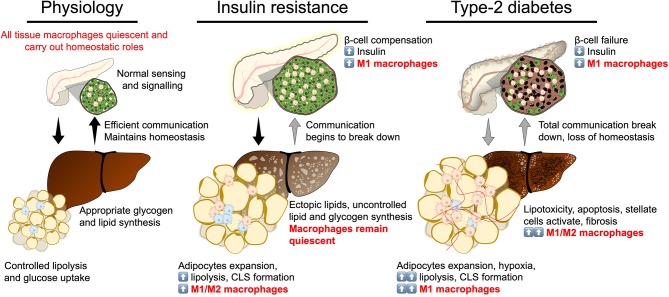
Break down of insulin secretion and sensitivity in type-2 diabetes. At the physiological state glycaemic homeostasis is maintained by efficient communication between the insulin secreting organ, the pancreas, and insulin target organs (adipose tissue and liver). All tissues are populated by their respective tissue macrophages that participate in maintaining tissue homeostasis and physiological function. Insulin resistance is a breakdown of communication at insulin target tissues. At the onset of insulin resistance, macrophages accumulate in adipose tissue and pancreatic islets. Crown-like structures develop in adipose tissue with heterogeneously polarized macrophages and a decrease in adipocyte insulin sensitivity. Pancreatic islet size increases with increased β-cell number and increased macrophages. Increase in β-cell number allows compensatory insulin release to overcome insulin resistance. Increasing insulin resistance and systemic inflammation result in β-cell failure when insulin secretion no longer compensates for resistance and persistent hyperglycaemia develops. At the stage of type-2 diabetes, a complete breakdown in inter-organ communication occurs, insulin secretion drops and inflammatory macrophages permeate adipose tissue and the liver. Chronic inflammation, hyperglycaemia and dyslipidemia lead to the development of non-alcoholic steatohepatitis.

Efficient communication between insulin secreting and insulin target tissues (the pancreas, adipose tissue, the liver, and skeletal muscle) maintains metabolic homeostasis in response to physiological challenges that transiently vary glycaemia or lipaemia, such as feeding or fasting (3–5). Insulin resistance represents a partial breakdown in communication between these tissues, where insulin target tissues become resistant to insulin signaling, despite initial compensation by the pancreas. T2D represents a stage of complete to near-complete breakdown of communication where production of insulin no longer meets the body's requirement to regulate glycaemia. Each of these tissues has its specialized niche of macrophages with important physiological functions maintaining tissue integrity, more importantly the tissue macrophage population undergoes adaptation at each stage of developing T2D (3–5, 17, 18). The tissue macrophage responses have been shown to be extremely powerful mediators of insulin signaling, sensitivity, and resistance (Figure 3).

### Pancreatic Islets Macrophages

Pancreatic islets, distributed within the exocrine pancreas, are micro-organs essential for systemic glucose homeostasis. β cells form the majority of the islet and respond to glucose, within seconds, by secreting the appropriate amount of insulin required for optimal energy supply to insulin-sensitive tissues. Innate immune cells also form part of the pancreatic islet. Under steady-state, macrophages are the major innate immune cell in both mice and humans (10, 19–21). Over 20 years after their discovery, islet macrophage phenotype remains unclear. Unlike ATMs and liver macrophages, islet macrophages do not adhere to the M2 vs. M1 polarization paradigm associated with metabolic protection and dysfunction, respectively. Indeed, M1 markers (CD11c, MHC-II) are constitutively expressed by macrophages in healthy islets, they also highly express IL-1β, TNF-α, and the pro-inflammatory transcription factor interferon regulatory factor (IRF)-5 (10, 19, 22). Moreover, they do not express M2 markers (CD206), in contrast to stromal macrophages of the exocrine pancreas (19).

The role of macrophages in islet homeostasis has only begun to draw attention. *In situ* islet imaging revealed that macrophages are in close contact with both β cells and vasculature, in mice (23). Islet macrophages monitor β cell insulin secretion in response to glucose by detecting endogenous ATP that is co-released with insulin (24). In turn, macrophages may also directly provoke or enhance insulin secretion through production of factors such as retinoic acid (10). Interestingly, relative to any other tissue, β cells have the highest expression of the signaling IL-1 receptor 1 (IL-1R1), strongly indicating a physiological role for IL-1β in β cell function (25, 26). It is well-established that acute, but not chronic, exposure to IL-1β stimulates insulin secretion in mice and humans (27, 28). Underlying mechanisms remain unclear, but may involve an increase in insulin granule docking at the plasma membrane allowing enhanced exocytosis (27). Two studies confirm this hypothesis with transgenic murine models. β cell-targeted deletion of IL-1R1 impairs peripheral glucose tolerance *via* reduced glucose-stimulated insulin secretion (29). Other studies report that feeding induces a physiological rise in circulating IL-1β, potentiating postprandial insulin secretion (30). IL-1β secretion was attributed to peritoneal macrophages responding to glucose metabolism and bacterial products, released IL-1β in-turn acts on β cells (30). It has not been ruled out that islet-resident macrophages may also produce IL-1β post-prandially, indeed these macrophages may be the main source of IL-1β in the islet microenvironment. Taken together, these previous reports show that physiological IL-1β levels play a critical role in amplifying insulin secretion.

During obesity, increased production of insulin is required to maintain normal blood glucose levels. As a result, the number of β cells and islet size increase, mainly by local proliferation of pre-existing β cells (Figure 3). Therein, macrophages slowly accumulate and may play an important role in β cell adaptation to early weight gain and the development of insulin resistance. In that context, islet macrophages may license β cell mass expansion and the required angiogenesis during the first weeks of high fat diet and in early islet adaption of young Db/Db mice. Indeed, macrophage-depleted mice showed lower β cell replication rate, decreased insulin secretion and impaired glucose tolerance compared to controls (31). The promotion of β cell proliferation by islet macrophages could be mediated by the platelet-derived growth factor receptor (PDGF-R) signaling pathway (32).

When obesity becomes chronic, insulin secretion eventually no longer compensates for increased insulin demands, resulting in hyperglycemia and T2D. This β cell failure is associated with local islet inflammation and production of inflammatory effectors (IL-1β, TNF-α, CCL-2) (20, 26, 32–37). This phenomenon is associated with increased macrophages in the islet in diet-induced or genetically obese rodents and in patients with T2D (20, 31–33, 35, 37). Two distinct subsets of macrophages have been identified in the islet: resident macrophages and pro-inflammatory macrophages. Islet-resident macrophages (CD11b^+^Ly6C^−^ or F4/80^high^CD11c^low^) predominate at steady-state and pro-inflammatory macrophages (CD11b^+^Ly6C^+^ or F4/80^low^CD11c^high^) accumulate during the course of obesity (32, 33). While CD11b^+^Ly6C^+^ macrophages are recruited from monocytes, F4/80^low^CD11c^high^ macrophages proliferate *in situ*. In this context, chlodronate liposome macrophage depletion rescues glucose-induced insulin secretion in models of genetic obesity and in palmitate-infused mice (33). Interestingly, despite increasing islet macrophage number, diet-induced obesity does not markedly alter macrophage phenotype (19, 32). Another source of inflammatory factors that may participate in islet inflammation are endocrine cells themselves, including β cells. Indeed, RNA sequencing of islet cells from T2D patients revealed an inflammatory signature associated with β cell dysfunction relative to islet cells from healthy controls, this result was attributed not only to immune cells but also to endocrine cells fuelling local inflammation (38, 39). These results, somewhat contradictory, suggest that islet macrophages are not solely responsible for islet inflammation in obesity. More studies are required to fully define their phenotypes and to investigate the roles that other innate immune cells may play, such as innate lymphoid cells (ILC) and their potential role in regulating insulin secretion and β cell mass expansion (10).

### Adipose Tissue Macrophages in Metabolic Homeostasis

AT is one of the first responders to alterations in energy balance. Physiologically AT regulates long term energy stores, appetite (through endocrine signaling) and body temperature (by providing insulation or even increasing thermogenesis in the case of brown adipose tissue). Adipose tissue macrophages (ATMs) generally present an M2 profile at steady state under physiological circumstances. They are characterized by expression of the mannose receptor CD206, CD301 alongside pan-macrophage markers such as F4/80 (in mice) CD14 (in humans) CD68 and CD11b. ATM homeostatic signaling includes expression of arginase 1 (ARG1), IL-10, and other type-2 effectors as well as catecholamines. The transcription factor peroxisome proliferator-activated receptor (PPAR)-γ is highly expressed in these cells and controls ATM oxidative metabolism and capacity to cope with a lipid-rich environment. In this niche, ATMs interact with other immune cells and provide signals for activation or repression of B and T cells, neutrophils, natural killer cells, and ILCs (40).

ATMs maintain tissue homeostasis by removing dying adipocytes and debris from dead cells; this efferocytotic process maintains an anti-inflammatory environment. Indeed, murine adipose tissue presenting an excessive rate of dying adipocytes due to targeted activation of caspase 8 are characterized by an increased number of alternatively activated anti-inflammatory macrophages (M2, CD206^+^), surrounding dead and dying adipocytes (41). This grouping of cells surrounding adipocytes in a ring-like structure are named crown-like structures (CLS). CLS are only occasionally found in lean AT. Under physiologic variation, AT homeostasis is challenged daily with periods of feeding, thus expansion and storage of lipids, or mobilization of stored-lipids during fasting or cold exposure. ATMs have enhanced lipid buffering capacities and this enables capturing lipids released from the dead adipocytes, also during physiological process such as weight loss, fasting-induced lipolysis (42), or thermogenesis (43). Interestingly, in obesity macrophage-mediated capture of excess lipids regulates systemic glucose tolerance. Lipids are stored within the macrophages and released into circulation in a controlled manner (44).

ATM lipid-buffering processes limit ectopic lipid storage, pro-inflammatory accumulation of lipids and systemic lipotoxicity/dyslipidemia. A program of lysosomal activity is activated in M2 ATMs to cope with environmental lipid overload. Interestingly, inhibition of lysosome biogenesis and consequently lipid accumulation and catabolism in ATMs decreases adipocyte lipolysis (45). More recently, novel pathways of lipid release independent of canonical lipolysis, have been described. Adipocytes release exosome-sized lipid-filled vesicles to be taken-up and stored by ATMs (46). The capture of lipids is facilitated by ATM expression of fatty acid transporter (CD36) and the lipid scavenger receptor MSR1 (45).

Much of the knowledge with regards to macrophage interactions with environmental lipids and their mechanisms of activation has come from the fields of atherosclerosis and the study of foam cells. Indeed, early studies carried out by Nagy et al. (47) brought to light the importance of such receptors as CD36, allowing macrophages to internalize oxidized lipids, which in turn act as nuclear receptor ligands (PPARγ in this case). The mechanisms described by Nagy et al. were amongst the earliest to elucidate links between metabolic stress, transcriptional regulation, and macrophage phenotypic plasticity.

The role of ATMs in thermogenesis is an emerging topic and pathways leading to the activating of ATMs are still under investigation (40). A novel population of macrophages involved in adipose tissue thermogenesis has been identified: sympathetic neuron associated macrophages (SAM) (48). These cells are morphologically different from ATMs and are located at fibers of the sympathetic nervous system in AT. Unlike ATMs, SAMs have the molecular machinery to uptake and catabolize norepinephrine which blunts catecholamine-induced lipolysis.

ATMs have also been associated with iron homeostasis, where intracellular iron is a source of free radicals and a cofactor for a number of proteins. Twenty-five percent of macrophages from lean adipose tissue are considered as ferromagnetic, i.e., iron-loaded and this proportion decreases with obesity (49). ATM iron recycling contributes to AT homeostasis, where an up-regulation of iron-related genes occurs during adipogenesis and an excess of iron contributes to adipocyte insulin resistance (50, 51).

ATMs play a more direct role in adipogenesis where alternatively activated macrophages form a niche for the development of adipocytes and in the vascularization of adipose tissue (52, 53). The accumulation of M2 ATMs in the CLS surrounding dead adipocytes leads to the recruitment of pre-adipocytes in response osteopontin (OPN). However, a recent study demonstrated that M2-like ATMs inhibit the proliferation of adipocyte progenitors through TGF-β signaling. A hallmark study by Buorlier et al. (53) characterized subcutaneous ATMs as being predominantly CD206^+^, and to be the major source of matrix degrading enzymes, making them an essential part of tissue remodeling. In this same study, secreted factors from ATMs were found to promote angiogenesis and inhibit adipogenesis in stromal-vascular fraction progenitor cells (53, 54). Controlling angiogenesis is a key factor in the maintenance of tissue homeostasis as it limits the formation of hypoxic areas and insures appropriate irrigation supplying nutrients and oxygen to the microenvironment. In the light of the above work, the physiological phenotype of ATMs can be largely seen as protective and may, in the early stages of caloric excess, act to coordinate adipose tissue adaptation (53).

Finally, alternatively activated ATMs are characterized by their production of IL-10, an anti-inflammatory cytokine known for its important role as a modulator of insulin sensitivity (55). Indeed, acute IL-10 treatment improves global insulin sensitivity *in vivo* (56) and its expression is positively correlated with insulin sensitivity in humans (57). Surprisingly, the hematopoietic deletion of IL-10 does not promote obesity nor insulin resistance, suggesting that other factors and pathways are involved in the maintenance of AT metabolic health (58). Furthermore, ATMs can release exosomes containing miRNA, such as miR-155, that regulate insulin sensitivity. Such ATM-derived exosomes from lean mice improve glucose intolerance and insulin sensitivity when delivered to obese mice (59).

### Adipose Tissue Macrophages and Metabolic Inflammation

Obesity is a complex pathology and a factor in the etiology of insulin resistance and T2D. The fundamental cause of obesity is chronic imbalance between energy expenditure and food intake leading to low-grade inflammation. Chronic low-grade inflammation is what is generally referred to when discussing metabolic inflammation, the starting point of which is the adipose tissue macrophage. An accumulation of inflammatory ATMs occurs in obesity and plays a key role in the pathogenesis of obesity-induced insulin resistance (Figure 3) (6, 7). Inflammatory ATMs correspond to the M1 subtype and are identified as F4/80^+^CD11b^+^ cells, also positive for CD11c and overexpressing IL-6, TNF-α, iNOS and the C-C chemokine receptor 2 (CCR2).

ATM accumulation in obesity occurs first due to *in situ* proliferation at CLS, and then by recruitment of circulating monocytes that differentiate into inflammatory macrophages (60). The first proliferative phase is driven by IL-4 signaling through Signal Transducer and Activator of Transcription (STAT)-6. Infiltrating macrophages increase upon CCL2 signaling to monocytes, several studies have demonstrated the importance of the CCR2/CCL2 axis in the recruitment of circulating monocytes (61). In addition, migratory capacity of macrophages is affected by obesity. Indeed, netrin-1, a laminin-related molecule known for its chemo-attractant/-repulsive properties, is induced by palmitate. It inhibits ATM migration to lymph nodes and consequently promotes ATM accumulation *in situ* (62).

The lipid-buffering capacity of ATMs is beneficial in early dysmetabolism and enhances a lysosomal program associated with M2 polarization (45), the abundance of lipids within ATMs impacts their polarization toward an M1 phenotype (63). Single-cell transcriptomic approaches confirm the heterogeneity of the ATMs, identifying three different macrophage populations in obese AT. Resident macrophages (F4/80^Lo^) expressing CD206 are maintained in obese AT, whereas Ly6c expression characterizes the newly recruited macrophages (also F4/80^Hi^). The pro-inflammatory subset of lipid-laden macrophages in CLS is characterized by the expression of CD9 (64). More recently, Jaitin and colleagues confirmed the phenotype and presence of CD9^+^ lipid-laden macrophages at CLS. They report that CD9^+^ cells counteract inflammation and adipocyte hypertrophy via the lipid receptor TREM2 (8). Proteomics analyses also identified specific ATM markers induced by stimuli reproducing the adipose tissue microenvironment with palmitate, insulin, and high levels of glucose (65). Such activation of ATMs gives rise to the metabolically activated macrophage (MMe), which is functionally and phenotypically distinct from classically activated M1 macrophages.

The importance of the pro-inflammatory capacity of the newly-recruited ATMs in the etiology of obesity is well established. Activated macrophages surround dead adipocytes and fuse to form multinucleate giant cells (66), an hallmark of chronic inflammation that correlates to insulin resistance (67). In 2008, Patsouris and colleagues demonstrated that the ablation of CD11c^+^ cells during obesity restored insulin sensitivity by decreasing inflammatory markers (68). Interferon regulatory factor IRF5 is a pro-inflammatory transcription factor, commonly restricted to CD11c^+^ cells, driving macrophage polarization toward an M1 phenotype (69), and is notably induced in ATMs in diet-induced obesity (70, 71).

### Liver Macrophages in Metabolic Homeostasis

Liver resident macrophages, also called Kupffer cells (KCs), represent up to 80–90% of the whole body macrophage population and are characterized by the expression of canonical macrophage markers (F4/80, CD14, CD68, CD11b) as well as the C-type Lectin (Clec)-4F (5). Clec4f is the marker of *bona fide* KCs that are functionally distinct, specialized and self-renewing tissue-resident macrophages (72). KCs belong to the reticuloendothelial system of the liver, they are located close to blood vessels in lumen of hepatic sinusoids, they regulate hepatocyte proliferation and apoptosis upon injury and at steady-state they clear blood of aged erythrocytes and recycle iron by degrading hemoglobin (73). Their location is adapted to their function of clearance of the portal blood flow from pathogens, micro-organisms and cellular debris (74). KCs select and eliminate debris from blood through scavenger receptors and canonical PRRs expressed on the cell surface. Importantly, KCs impose immune tolerance in the liver, an organ constantly exposed to antigens and bacterial endotoxins from the intestine and portal blood. KCs maintain an anti-inflammatory environment by several mechanisms, secretion of IL-10, low expression of MHC-II and high expression of PDL-1, limiting antigen-presentation capacity and a powerfully inhibiting T-cells, respectively (75). Interestingly, even upon IFN-γ priming, KCs promote differentiation of T_Regs_, a specialized immunoregulatory subset of T-cells that maintains immune tolerance (75, 76). At steady-state, KCs have limited interactions with distant non-immune cell types, because they are not typically motile cells. When microenvironmental communication is required, KCs secrete cytokines or signal to circulating monocytes to differentiate *in situ* (73).

### Liver Macrophages in Metabolic Inflammation

Systemic extension of inflammation from AT is associated an increase of pro-inflammatory mediators in circulation and an increase in adiposity. Insulin resistance, persistent glucolipotoxicity, and systemic inflammation coincide in ectopic fat deposition, a major site of which is the liver. In obesity and T2D, the liver undergoes a spectrum of changes that range from benign steatosis to fibrosis and cirrhosis (77). This range of pathologies is known as non-alcoholic fatty liver disease (NAFLD), where lipotoxicity, inflammation and fibrogenesis characterize the more advanced stages of non-alcoholic steatohepatitis (NASH). Liver macrophages, KCs, are key actors in the progression of NASH, due to their pro-apoptotic and pro-inflammatory responses to lipotoxic hepatocytes and their capacity to activate matrix producing hepatic stellate cells (HSCs).

Ectopic fat deposition triggers activation of immune cells and an inflammatory environment which favors insulin resistance. Surprisingly, unbiased transcriptomic analysis revealed no differences in terms of expression of genes associated with a pro-inflammatory signature, between liver macrophages from lean and obese patients (similar data were obtained from mice fed an HFD for 9 weeks). Metabolic impairments are not associated with a pro-inflammatory activation of liver macrophages (78). However, the transcriptomic inflammatory signature is indeed variant between the stages of benign steatosis and NASH. At the transition between steatosis and NASH, liver macrophages target lipotoxic hepatocytes inducing their apoptosis and signal to HSCs to induce their activation (77). Chronic insults on the liver will result in fibrosis as an exuberant scarring response to dead or dying hepatocytes, sustained fibrogenesis will in-turn affect liver function (77). Interestingly at the NASH stage, the liver macrophage pool is extremely heterogenous, with M1-like macrophages inducing hepatocyte apoptosis and M2-like macrophages promoting HSC activation and fibrogenesis (77, 79).

The pro-inflammatory transcription factor IRF5 has been shown to play a critical role in liver macrophages, mediating the transition between benign steatosis and NASH. Blunting IRF5 expression results in hepatoprotection through early upregulation of anti-apoptotic and immunoregulatory signaling, increasing T_Reg_ differentiation and IL-10 secretion upon hepatocellular stress (79). Recent research is delving into potential non-inflammatory or non-immune signaling efferent from KCs, notably effector molecules such as insulin-like growth factor-binding protein (IGFBP)-7, regulates insulin sensitivity in the context of obesity (80).

## Initiating and Sustaining Macrophage Polarization in T2D

Defining the extracellular metabolic and molecular signals associated with macrophage polarization in metabolic inflammation and insulin resistance is an area of active research. Candidate “metabolic” immunogens include lipids, hypoxia, cell death, and stress (42, 66, 81).

Ninety percent of ATMs are surrounding dead adipocytes in fat depots of genetically obese mice (82) suggesting that dead adipocytes are sources of DAMPs that lead to CLS formation and/or the accumulation of ATMs. Obese AT is also characterized by hypoxic areas and the expression of hypoxia-related genes, including HIF-1α. This transcription factor also promotes the pro-inflammatory capacities of ATMs in the context of obesity (83). Furthermore, lipolysis products and more generally lipids whose circulating levels are elevated in obesity, are extremely attractive candidates for the induction of an inflammatory response in ATMs. TLR-4 has been shown to be activated by nutritional fatty acids in macrophages, inducing pro-inflammatory signaling pathways (84). Macrophages can be activated by triglyceride-rich lipids, such as palmitate or very-low density lipoproteins (VLDL) which upregulate intracellular levels of ceramides and potentiate the pro-inflammatory response (85). Activation of the NLRP3-inflammasome by these mechanisms induces caspase-1-mediated cleavage of pro-IL-1β and pro-IL-18 into their active forms. Interestingly, saturated fatty acids such as palmitate have been shown to activate the NLRP3-inflammasome through an AMPK-autophagy-mitochondrial ROS signaling axis, leading to secretion of IL-1β and IL-18 (86). Importantly, IL-1β secretion *per se* is associated with insulin resistance. Indeed, IL-1β prevents insulin signaling through TNF-α-dependent and independent mechanisms (87). Once established, this pro-inflammatory environment favors the production of pro-inflammatory cytokines recruiting monocytes and other immune cells that sustain low-grade chronic inflammation.

Pro-inflammatory cytokines are key actors of the disruption of insulin signaling leading to insulin resistance (88). They act through paracrine mechanisms on insulin sensitive cells such as adipocytes. Physiologically, upon insulin binding to its receptor, the phosphorylation of tyrosine residues of insulin receptor substrate (IRS)-1 activates intracellular signaling pathways mediating insulin action (89). In the context of metabolic inflammation, JNK-1 and IKK are capable of interfering with insulin signaling by phosphorylating inhibitory serine/threonine residues of IRS-1. Insulin signaling is therefore disrupted (90). Similar pathways involving JNK-1 and IKK can be activated through the binding of fatty acids to TLRs. Moreover, IL-1β, which also signals through IKKβ and NFκB, favors insulin resistance by repressing IRS-1 expression at both transcriptional and post-transcriptional levels (91). Interestingly, IL-6 signaling inhibits insulin sensitivity through distinct mechanisms involving the JAK-STAT pathway that controls the transcription of its own suppressor, known as suppressors of cytokine signaling (SOCS), notably SOCS3. High levels of circulating IL6 induce increased expression of SOCS3 which physically interacts with tyrosine phosphorylated residues, and consequently inhibits IRS-1 binding to the insulin receptor (92).

## Metabolic Mechanisms of Macrophage Polarization

**Figure 4:**
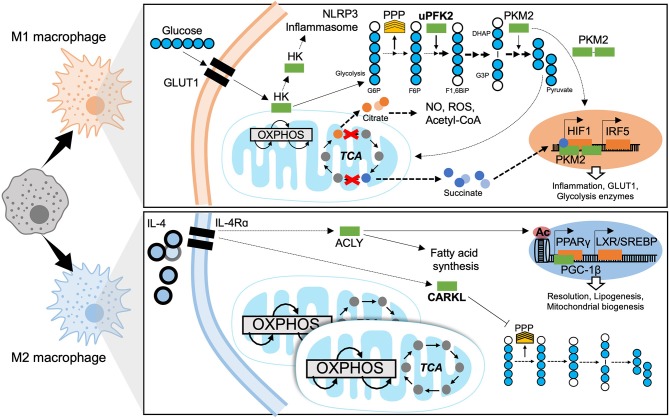
Metabolic mechanisms of macrophage polarization. M1 macrophages are characterized by predominantly glycolytic metabolism. Glycolysis consists of breaking down a 6-carbon glucose molecule (where each carbon is depicted as a blue circle, white when phosphorylated) into 3-carbon sugars then into pyruvate, ATP, NADH, and H^+^. The transcriptional programme that supports glycolysis is mediated by HIF1 and at least in part by IRF5. A Glucose substrate is provided by increased expression of the glucose transporter GLUT1. Meanwhile several glycolytic enzymes undertake non-canonical roles to support M1 effector functions. The mitochondrial tricarboxylic acid (TCA) cycle is disrupted, leading to accumulation of citrate and succinate which also enhance M1 effector function. The M2 macrophage has a fully intact TCA cycle, enhanced OXPHOS and increased mitochondrial biogenesis. ATP citrate lyase (ACLY) is activated downstream of IL4 signaling and enhances M2 effector functions through epigenetic mechanisms and producing substrates for lipogenesis. The sedoheptulose kinase (CARKL) represses the pentose phosphate pathway (PPP). Transcriptional programmes for M2 macrophage metabolism are mediated by PPARγ and LXR. GLUT1, Glucose transporter-1; HK, Hexokinase; NLRP3, NACHT, LRR, and PYD domains-containing protein; OXPHOS, oxidative phosphorylation; TCA, tricarboxylic acid cycle; PPP, pentose phosphate pathway; uPFK2, ubiquitous phosphofructokinase2; PKM2, pyruvate kinase isozyme 2; G6P, glucose-6-phosphate; F6P, fructose-6-phosphate; F1,6BiP, Fructose-1,6-biphosphate; G3P, glyceraldehyde-3-phosphate; DHAP, dihydroxyacetone phosphate; NO, nitrous oxide; ROS, reactive oxygen species; CoA, Coenzyme A; HIF1, hypoxia-inducible factor 1; IRF5, interferon regulatory factor 5; IL-4, interleukin 4; IL-4Rα, IL-4 receptor alpha; ACLY, ATP-citrate lyase; CARKL, carbohydrate kinase like/sedoheptulose kinase; Ac, acetylation mark; PPARγ, peroxisome proliferator-activated receptor gamma; LXR, liver X receptor; SREBP, sterol regulatory element binding protein; PGC-1β, PPARγ coactivator 1-beta.

As with any other cell, macrophages have their own metabolic requirements and depend on the same well-characterized bioenergetic pathways as non-immune cells; these pathways are broadly classified into glycolytic or mitochondrial (Figure 4). In addition to pro-inflammatory signaling and transcriptional control, cellular metabolism is gaining recognition for the key role it plays in macrophage terminal differentiation. Mobilizing metabolic pathways does not solely produce energy but also dictates the magnitude of macrophage effector function (13). Early studies in immunometabolism characterized fundamental mechanisms fuelling macrophage function in model systems with canonical activators. Such foundation studies allowed clear association of bioenergetic profiles to polarization states. Current research is expanding on these paradigms through investigating bioenergetic profiles and metabolic adaptation of tissue-specific macrophage niches under physiological and pathological conditions and in response to diverse stimuli. Interestingly, the metabolic classification of macrophages was one of the first to be made, with the initial observation that M2 macrophages are able to metabolize arginine (93).

### Metabolic Adaptation of Pro-inflammatory Macrophages

The enhanced glycolytic activity of the pro-inflammatory macrophages was observed decades ago (94) but the mechanisms underlying this process and its physiological significance were only recently described. It is a hallmark metabolic response in the polarization of macrophages toward an M1 phenotype (Figure 4). Glycolysis corresponds to the metabolic pathway responsible for the conversion of glucose into pyruvate, through 10 sequential enzyme-catalyzed reactions. This pathway gives rise to the production of ATP and NADH.

Glycolytic metabolism facilitates pro-inflammatory differentiation to enable efficient bacterial killing (95) and the secretion of pro-inflammatory mediators. Experimental inhibition of glycolysis with 2-deoxy-glucose (2-DG) limits the pro-inflammatory macrophage response to LPS (96). The rapid induction of glycolysis is enhanced by the upregulation of glucose transporter (GLUT)-1 expression (97). The switch toward glycolytic metabolism is dependent on the transcription factor HIF-1α (98). Its stabilization in hypoxic conditions promotes anaerobic metabolism and enhanced transcription of genes encoding glycolytic enzymes, such as pyruvate dehydrogenase kinase (PDK) and hexokinase (HK) which catalyse glucose phosphorylation. By HIF-1α-independent mechanisms, the ubiquitous isoform of phosphofructokinase-2 (uPFK2) is induced in M1 macrophages. Whilst uPFK2 is a more active isoform of PFK2, its induction enhances glycolytic flux and favors the formation of fructose-2,6P_2_ which allosterically activates PFK1, the enzymes catalyzing commitment to glycolysis (99). As well as HIF-1α-dependent mechanisms, studies of IRF5 risk-variants report that gain-of-function single nucleotide polymorphisms of IRF5 (associated with auto-immune disease) increase glycolysis and inflammatory signaling, basally and in response to LPS (100).

Some glycolytic enzymes have non-canonical roles in macrophages. Notably, pyruvate kinase isoenzyme 2 (PKM2), induced by LPS (101), can be found as a dimer. This dimer can translocate to nuclei and act as a coactivator for HIF-1 (Figure 4). Consequently, PKM2 participates in a positive feedback loop with the up-regulation of pro-inflammatory and glycolytic genes in response to HIF-1 activation (102). Moreover, HK1 can be inhibited by bacterial products and then dissociate from the mitochondria, which activates the NLRP3 inflammasome and the downstream production of pro-inflammatory cytokines (103). Mechanisms of resolution of glycolytic programming have not yet been brought to light; however, a recent study by Ip et al. demonstrates that IL-10 signaling exerts its anti-inflammatory effects by inhibiting the translocation of GLUT1 to the membrane (104). As well as being a substrate for glycolysis, glucose also fuels the pentose phosphate pathway (PPP), required for the synthesis of nucleotides and NADPH destined for ROS production by NADPH oxidase. The PPP is also induced upon LPS stimulation and M1 polarization (105).

The Krebs/tricarboxylic-acid (TCA) cycle is a mitochondrial metabolic pathway enabling ATP production and provision of substrates for the electron transport chain (ETC) that supports oxidative phosphorylation (OXPHOS) (Figure 4). In the context of pro-inflammatory macrophages, the TCA cycle is disrupted at two key steps: (i) accumulation of citrate due to a decrease in isocitrate lyase expression and (ii) the accumulation of succinate. Mitochondrial efflux of citrate is enhanced in M1 macrophages. Citrate accumulation has functional relevance to inflammatory polarization, being required for the production of ROS, NO, and prostaglandins (106). Citrate also acts a substrate for transformation into acetyl-CoA, feeding fatty acid synthesis through the ATP-citrate lyase (ACLY) (107). Interestingly, inhibiting fatty acid synthesis by silencing fatty acid synthase (FAS) in myeloid cells, has been shown protective in diet-induced insulin resistance, hindering ATM recruitment and chronic inflammation in mice. This underlies the importance of lipid metabolism in the polarization and function of macrophages, and notably synthesis and composition of the plasma membrane (108). Finally, the accumulation of citrate leads to a decrease in the levels of cis-aconitate which is the precursor of itaconate, a well-described anti-inflammatory intermediate. Itaconate exerts its anti-inflammatory effects by inhibiting succinate dehydrogenase (SDH), ROS production, and the release of pro-inflammatory cytokines.

An itaconate negative feedback loop has been described in the context of LPS and IFNγ stimulation, where itaconate shuts down the inflammatory response (109, 110). On the other side, the accumulation of succinate favors SDH activity and production of mitochondrial ROS (111). Succinate can trigger the expression of IL-1β through stabilizing HIF-1α (112). Consequently, pro-inflammatory macrophages are characterized by an increase of glycolytic activity and decreased OXPHOS. Interestingly, acute LPS treatment induces a burst of oxidative metabolism in macrophages which increases the pool of available of acetyl-CoA. This process supports histone acetylation and the downstream transcription of pro-inflammatory genes (113). The shutdown of oxidative metabolism, a hallmark of M1 macrophages, occurs following longer LPS treatments.

Finally, amino acids, the immunometabolism of which is relatively less known, can also be metabolized and influence macrophage polarization. For example, glutamine catabolism feeds the TCA cycle by giving rise to α-ketoglutarate, which acts as a co-factor for histone modifying enzymes implicated in macrophage differentiation (114). Arginine is also metabolized into L-citrulline simultaneously to the production of NO by iNOS, favoring the killing of bacteria.

### Metabolic Adaptation of Anti-inflammatory Macrophages

Mitochondrial respiration dominates the M2 polarized state. M2 macrophages are characterized by an intact, fully functional TCA cycle and enhanced OXPHOS (Figure 4). Fatty acid oxidation (FAO) and mitochondrial biogenesis are increased in a PPAR-γ-coactivator-1β (PGC-1β)-dependent manner (115). With FAO being the main source of substrates, glycolysis-fuelled OXPHOS is not required to maintain the M2 phenotype (116).

The molecular mechanisms linking the metabolic adaptations of M2 macrophages to their functions in tissue homeostasis remain largely unexplored. Interestingly, IL-4 is known to activate ACLY enhancing substrate formation for histone acetylation. This epigenetic modification enables the transcription of specific M2-genes (117). Other proposed mechanisms implicate the carbohydrate kinase-like protein (CARKL), a sedoheptulose kinase that regulates PPP. CARKL is down-regulated in response to LPS and highly expressed upon IL-4 stimulation (118). CARKL activity inhibits the PPP in the M2 state (Figure 4).

Glutamine metabolism also plays an important role in M2 polarization. The expression of Slc1a5, a glutamine transporter, is increased upon IL-4 stimulation (119). Glutamine catabolism, in addition to glucose metabolism, leads to the formation of UDP-GlcNAc that supports N-glycosylation, a process required for the expression of several M2 markers (120).

Lipid synthesis, mediated by LXR, is central to M2 effector function and resolution of inflammation (121). Upon pro-inflammatory activation, LXR-dependent lipogenesis is inhibited. LXR being a the pro-lipogenic nuclear receptor and transcription factor later engages the master regulator of lipogenesis, SREBP1 to mediate the production of anti-inflammatory lipids (i.e., eicosanoids, resolvins) (122).

### Deciphering Metabolic Adaptations of Tissue Resident Macrophages and Insulin Resistance

The above fundamental findings in macrophage bioenergetics were largely established using *ex vivo* modeling systems (such as murine bone marrow- or human monocyte-derived macrophages) and in response to known polarizing agents. Whilst these mechanisms apply to a large proportion of macrophages, typically infiltrating macrophages, responses to complex metabolic stimuli and the heterogeneity of tissue resident macrophages remains to be addressed. Tissue-resident macrophages face nutrient competition, normoxic and hypoxic areas and interactions with other cells. They respond to complex stimuli rather than unique stimuli. The bioenergetic adaptations of tissue-resident macrophages in obesity and insulin resistance remain to be thoroughly elucidated.

Interestingly, ATMs in obesity have a unique hypermetabolic profile with both increased glycolysis and OXPHOS compared to lean ATMs, whilst maintaining a pro-inflammatory phenotype (123). More precisely, the pro-inflammatory capacity of the obese ATMs is mediated by glycolysis independently of HIF-1α (123). This bioenergetic profile is also distinct from peritoneal macrophages, despite the shared systemic glucolipotoxicity brought on by obesity. These observations underlie the specificity of metabolically activated macrophages and ATMs.

Hypoxic areas develop in AT upon inappropriate expansion in obesity and insulin resistance. Hypoxia and inadequate angiogenesis are attractive mechanisms leading to macrophage metabolic activation and their inflammatory polarization. Alternatively, the abundance of free fatty acids or lipolysis products in adipose tissue makes for a nutrient-/substrate-rich microenvironment. The effect of such lipid loading on macrophage metabolism and polarization remains to be investigated under iso- or hyper-caloric conditions. For example, the effect of obesity on macrophage glutamine metabolism remains to be investigated. Glutaminolysis is decreased in the AT of obese patients compared to lean subjects and glutamine levels in serum are decreased in patients with obesity or diabetes, suggesting an influential role for glutamine metabolism in ATM polarization (124).

## Transcriptional Control of Macrophage Polarization

**Figure 5:**
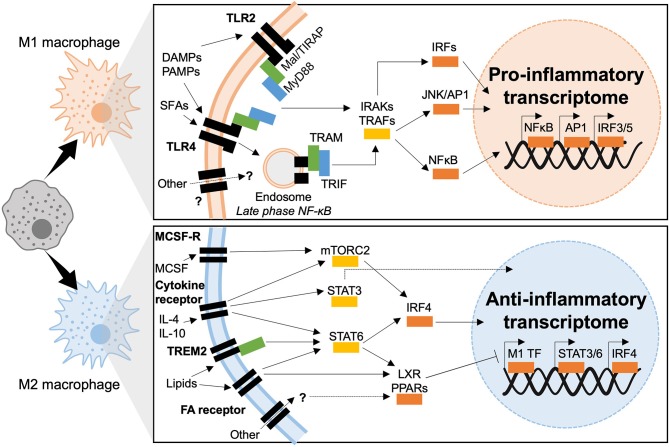
Transcriptional mechanisms of macrophage polarization in T2D. M1 macrophages polarize in response to TLR2 or TLR4 ligation, downstream signaling is dependent of two adaptor complexes: Mal/TIRAP–MyD88 (TLR2 and TLR4) and TRAM–TRIF (TLR4). IRAK and TRAF signaling then dictate which transcriptional programmes are engaged, IRF3 IRF5, AP1, and NFκB have been shown to induce inflammatory polarization in T2D. M2 macrophages polarize downstream of cytokine and fatty acid receptor stimulation (also possible through other molecules such as TREM2). Intermediate signaling is largely through STATs and nuclear receptors (LXR and PPAR). STATs act as transcription factors or activate IRF4, whereas nuclear receptors act through transrepression of M1 transcription factors (M1 TF).

Transcriptional control of macrophage polarization is well-characterized downstream of TLR ligation. Hallmark studies identified major TLR ligands as well as the key transcription factors that mediate inflammatory responses. Many of these pathways have been investigated in metabolic disease and are key mediators of macrophage activation in obesity, insulin resistance and T2D (Figure 5).

### TLR-Dependent Inflammation in T2D

TLRs are highly-conserved transmembrane receptors expressed in and on macrophages. Their conservation is attributed to the evolutionary requirement to recognize structurally conserved molecules and pathogens (125). Each TLR, from TLR1 to TLR13 recognizes specific ligands ranging from LPS, to nucleic acids, viral particles and chitin. Alongside their canonical roles in host-defense, several TLRs are implicated in metabolic inflammation and insulin resistance (126, 127). In this light, TLRs recognize not only infectious pathogens (through PAMPs) but also metabolic stressors or DAMPs associated with sterile inflammation and glucolipotoxicity.

The main TLRs implicated in diabetogenesis are TLR2 and TLR4. Engaging these two TLRs gives rise to chronic inflammation and insulin resistance through direct interference with insulin signaling (127–129). In macrophages TLRs 2 and 4 share common adaptor proteins, the myeloid differentiation primary response (MyD88) protein and Mal/TIRAP, that recruit IRAK kinases upon TLR engagement and dimerization. IRAK 1, 2, and 4 downstream signaling activates NFκB and Activator Protein (AP)-1. TLR4 also activates other downstream signaling. It is the only TLR that forms complexes with all adaptor proteins, Mal/TIRAP and MyD88, to initiate the early-phase NFκB response, the complex is then endocytosed and endosomal TLRs associate with TRAM and TRIF adaptors. Canonically, TRAM and TRIF set in motion the type-1 interferon response, transcriptionally mediated by Interferon Regulatory Factors (IRFs), AP-1 and late-phase NFκB activation. Both early and late phase action is required to sustain production of inflammatory cytokines (127–129). Co-ordinated action of TLRs, adaptor proteins and kinases result in the sustained activation of three major transcriptional programmes, headed by IRFs, AP-1, NFκB, and JAK-STAT.

### Interferon Regulatory Factors

Initially characterized for their binding to virus-inducible enhancer elements on interferon coding regions, interferon regulatory factors (IRFs) are renowned for their control over innate immunity and type-1 interferon signaling. Also forming part of JAK-STAT signaling, IRFs respond to a number of DAMPs and PAMPs, mediate sterile inflammation (metabolic and auto-immune) and are also active in non-immune cells (e.g., adipocytes) (130, 131).

IRF family members are 300–500 amino acids long, share a conserved N-terminal DNA binding domain allowing binding to interferon sensitive regulatory elements. The C-terminal IRF association domain is variable and allows dimerization between the different IRFs (132). IRFs 1–5 and IRF9 control macrophage differentiation and polarization in response to PRR ligands, IRFs 3, 4, and 5 have been reported to play a role in metabolic inflammation (131).

IRF5 is responsible for M1 macrophage polarization, it is implicated in sterile inflammation and auto-immunity, namely rheumatoid arthritis where risk-variants contributing to the over-expression of IRF5 have been reported (131). In T2D, IRF5 contributes to macrophage activation and metabolic decline in both adipose tissue and in the liver.

In ATMs, IRF5 is highly expressed by CD11c^+^ macrophages at CLS. Both CLS formation and IRF5 expression are strongly associated with AT inflammation, maladaptive adipocyte expansion and both local and systemic insulin resistance (70). Upon diet-induced obesity, mice with a myeloid-deficiency of IRF5 remain insulin sensitive despite increased adiposity. Visceral white adipose tissue in IRF5-deficiency is characterized by adaptive remodeling mediated by a *de facto* type-2 immune response, limiting adipocyte expansion and preventing loss of sensitivity to insulin's anti-lipolytic effect (70). Dysregulated expression of IRF5 is also causal in the progression to NASH. Throughout NAFLD, IRF5 mediates pro-apoptotic and inflammatory signaling from liver macrophages toward lipotoxic hepatocytes. Sustained inflammatory signaling and hepatocyte apoptosis result in scarring fibrogenesis in the liver (79).

IRF5 is the active transcription factor canonically downstream of TLR4. Interestingly, although the phenotypes of TLR4-deficiency and IRF5-deficiency are near identical under diet-induced obesity, the TLR4-IRF5 axis remains to be experimentally confirmed in the pathogenesis of T2D and its complications (133). Similarly downstream of TLR4, IRF3 promotes AT inflammation upon diet-induced obesity and inhibits adipose tissue browning. IRF3-deficient mice retain insulin sensitivity upon high-fat feeding and enhance AT browning (134).

In opposition to IRF3 and IRF5, IRF4 promotes macrophage M2 polarization and the resolution of inflammation (135). The metabolic phenotype observed in IRF4-deficient mice on HFD is accordingly aggravated (136). Myeloid-deficiency of IRF4 results in increased insulin resistance and adipose tissue inflammation when compared to IRF4-competent mice (136). Interestingly, IRF4 is nutritionally regulated by insulin signaling and by canonical transcription factors involved in metabolic signaling (e.g., FOXO1). Additionally, IRF4 regulates lipid handling in adipocytes, promoting lipolysis by facilitating lipase expression (130).

### Activator Protein 1

AP-1 is a complex formed of the proto-oncogenes c-Jun and c-Fos that are essential for DNA binding. AP-1 activation responds to cytokine signaling and growth factors; it controls apoptosis, cell growth, and macrophage terminal differentiation to an M1-like phenotype (137).

AP-1 activity is dictated by post-translational modifications, notably translocation and/or dimerization of its subunits, by signaling from c-Jun N-terminal (JNK) and mitogen-activated protein kinases (MAPK). AP-1 activity is also regulated by the composition of its DNA binding dimer (Jun/Jun, Jun/Fos, bZIP) and through binding partners (138). AP-1 is canonically activated in response to PRR ligation, cytokine signaling and growth factors. In the case of metabolic inflammation AP-1 is responsive to saturated fatty acids (SFAs), namely palmitate (128). Macrophages exposed to palmitate release pro-inflammatory mediators in an AP-1 dependent manner (128).

AP-1 activity is also responsive in response to hormone signaling, where leptin increases binding of nuclear proteins to the AP-1 consensus sequence of the lipoprotein lipase (LPL) gene promoter. This activity increases macrophages expression of LPL, giving mechanistic insight into the role of AP-1 in foam cell formation, atherogenesis and T2D (139).

The upstream kinases that activate AP-1 subunits have been extensively investigated in metabolic disease, namely JNKs. Mice-deficient for JNK1 and/or JNK2 remain metabolically healthy upon diet-induced obesity, mice gain less weight, are protected from insulin resistance and inflammation (140, 141). Interestingly, myeloid-specific deficiency of JNK, results in non-inflammatory obesity and a decrease in serum fatty acids. Studies indicate that myeloid-AP-1 is a key mediator of adipose tissue lipolysis upon diet-induced obesity (142).

### Nuclear Factor-κB

NFκB is a transcription factor that promotes M1 polarization, it responds to a variety stress signals including: cytokines, redox stress, oxidized lipids, bacterial, or viral antigens (143–146). Dysregulated NFκB signaling occurs in a number of inflammatory conditions including T2D. NFκB is highly expressed in ATMs upon their M1/MMe differentiation and throughout the onset of insulin resistance. Furthermore, cytokines released by M1/MMe macrophages form an amplifying loop that recruits and polarizes other leukocytes at the site of inflammation.

Mice with a myeloid-deficiency of *Inhibitor of NF*κ*B Kinase* (IKK-β), NFκB's canonical activator protein, display a diminished inflammatory response in diet-induced obesity and maintain systemic insulin sensitivity (147). Interestingly, hepatic deficiency of IKK-β only retains insulin sensitivity in the liver (not in muscle nor AT), indicating that the myeloid-derived IKK-β/NFκB is the main regulator of systemic metabolic homeostasis (147).

### Signal Transducers and Activators of Transcription

A family of 7 transcription factors that regulate interferon signaling, Signal Transducers and Activators of Transcription (STATs), have well-established roles in apoptosis, proliferation, and differentiation of innate immune cells. Of note, STAT activity is particularly important in maintaining immune tolerance. STATs are activated downstream of cytokine, chemokine, and growth factor signaling. STAT dimerization and nuclear translocation is dependent on phosphorylation mediated Janus Kinase (JAK), together forming the JAK-STAT pathway.

STATs 1 and 5 promote M1-like signaling whereas STATs 3 and 6 promote M2-like signaling in macrophages (148–152). Interestingly the more recently described Mme phenotype is polarized independently of STAT1 activity (153).

STAT1 in macrophages is activated in response to high glucose and exerts pro-inflammatory signaling through epigenetic mechanisms. Of note, glucose-responsiveness of STAT1 has been reported in *in vitro* and *ex vivo* modeling, with little-to-no evidence being reported *in vivo* or from human studies of obesity, insulin resistance, and T2D (154, 155). To date no evidence links STAT5 activation *per se* to diabetic pathogenesis despite its known roles in inflammatory polarization.

STAT3 is strongly linked to the development of T2D and its complications, mainly with anti-inflammatory, metabolically protective properties. For example, STAT3 is a downstream target of the first-line T2D treatment, metformin. Metformin inhibits the differentiation of monocytes to macrophages and decreases their infiltration into atherosclerotic plaques through AMPK-mediated inhibition of STAT3 (156). Similarly, in insulin resistance and diet-induced obesity, protective effects of ABCA1/APOA1 activity are STAT3-dependent, as is the anti-inflammatory adipose tissue phenotype of mice with a myeloid-deficiency of JAK2 (157, 158).

STAT6, on its own, or in concert with the vasodilator-stimulated phosphoprotein (VASP) has immunoregulatory properties in the context of metabolic inflammation. The VASP-STAT axis has been described in mice with a myeloid-specific deficiency of VASP, mice were prone to hepatic inflammation and insulin resistance in a STAT6-dependent manner (159). Whereas, STAT6 deficiency predisposes mice to diet-induced obesity, oxidative stress, and adipose tissue inflammation (160).

### Peroxisome Proliferator-Activated Receptors (PPARs)

PPARα, γ, and δ/β, are expressed at different levels in different tissues and vary across developmental stages. Highest expression levels are in the liver, skeletal, and cardiac muscle and in the spleen. PPARs are implicated in cellular metabolism, differentiation, development and more recently emerged as key regulators of inflammation.

In M1 macrophages PPAR-α inhibits the expression of pro-inflammatory mediators by negative regulation of AP-1 and NFκB. Several studies report the beneficial effects of PPAR-α activation in T2D and its complications. PPAR-α agonists have been applied in T2D patients and are beneficial in atherosclerosis, through inhibiting foam cell formation and inflammatory signaling. Beneficial effects are mediated by interfering with c-Fos and c-Jun interactions and by limiting lipid accumulation through repressing Fatty Acid Transport Protein (FATP)-1 (161–163).

PPAR-β*/*δ also acts on macrophage metabolism, regulating lipid efflux, fatty acid catabolism and beta-oxidation. PPAR-β*/*δ in macrophage regulates whole body energy dissipation and systemic responses to cholesterol; PPAR-β*/*δ activation occurs in response to dyslipidemia (164–166). In the pathogenesis of T2D, PPAR-β/δ plays a protective role controlling macrophage infiltration in adipose tissue and liver and promoting immune tolerance (M2 polarization) in ATMs acting downstream of STAT6 (167). Mice with a myeloid-deficiency of PPAR-β*/*δ display an aggravated metabolic phenotype upon diet-induced obesity.

PPAR-γ plays an important role in adipose physiology, adipocyte differentiation and maturation. Of the two known isoforms, PPAR-γ1 is expressed in macrophages and adipocytes whilst PPAR-γ2 is restricted to adipocytes (168). PPAR-γ1 enhances monocyte differentiation into M2 macrophages and is an inhibitor of inflammatory polarization, repressing MMP9, IL-6, TNF-α, and IL-1β expression (161, 169, 170). In *in vitro* and *ex vivo* modeling, PPAR-γ inhibits M1 signaling associated with LPS+IFNγ stimulation, including iNOS, COX-2, and IL-12 (171–173). Importantly, macrophage PPAR-γ is also a downstream target of internalized lipids, and mediates expression scavenger receptors required for foam cell formation (47). Accordingly, PPAR-γ-deficient mice display impaired M2 maturation and develop exacerbated insulin-resistance and metabolic inflammation in diet-induced obesity (174, 175). Enhancing PPAR-γ activity with thiazolidinediones (TZDs) improves the metabolic phenotype in diet-induced obesity (176). Interestingly, reports of PPAR-γ overexpression demonstrate that mature adipocyte PPAR-γ is in-fact the main insulin-sensitizing component (overexpression phenotype is comparable to TZD treatment) (177). Little-to-no beneficial effects are observed upon diet-induced obesity when PPAR-γ is overexpressed in macrophage (177). Such over-/under-expression studies reveal divergent functions of PPAR-γ. Further mechanistic work is needed to precisely characterize the roles and regulation of this nuclear receptor and its different isoforms in different cell types and microenvironments.

With regards to mechanism of action, multiple mechanisms have been proposed, with the main one being *transrepression*, whereby PPAR-γ binds to active pro-inflammatory transcription factors and represses their function. Repressive mechanisms through interactions with nuclear receptor corepressor (NCoR) complexes have also been proposed (178).

### Liver X Receptors

Liver X receptors (LXRs) exist in 2 isoforms, LXRα and LXRβ, both of which are lipid-activated and regulate macrophage inflammatory responses. To regulate transcription LXRs heterodimerise with Retinoid X Receptor (RXR) and bind to LXR response elements on the genome (179). LXRs, play important roles in T2D and in cardiovascular disease, promoting anti-inflammatory polarization and regulating macrophage lipid content.

LXR activation by oxysterol species and synthetic compounds allows cholesterol efflux from macrophages through the lipid transporters ACBCA1 and ACG1 (180). LXRs also directly repress transcription of pro-inflammatory genes and enhance transcription of anti-inflammatory genes in response to polarizing stimuli (181). Mechanistically, LXRs exert their effects by transrepression once they are sumoylated. This modification prevents LPS-dependent exchange of corepressors, thus maintaining LXR-mediated repression of inflammatory transcription factor activity (182). Several reports show the protective roles that LXRs have in metabolic inflammation and insulin resistance. Namely, LXR agonists act as insulin sensitizers and regulators of glycaemia through repressing hepatic gluconeogenesis (183–185).

### Hypoxia Inducible Factor 1

Hypoxia inducible factor (HIF)-1 is a transcription factor with two subunits, α and β. HIF-1α is stabilized and its expression is increased in response to hypoxia, whereas HIF-1β is constitutively expressed and stabilized independently of oxygen levels (186). Under hypoxic conditions, HIF-1α translocates to the nucleus and dimerises with HIF-1β allowing binding to hypoxia response elements (HREs) on the genome and regulation of target gene expression (187). Under oxygen-poor conditions, HIF1 activation mediates a shift toward anaerobic respiration in cells where bioenergetic requirements are supported by glucose metabolism (188).

Myeloid-specific overexpression of HIF-1α leads to increased M1 polarization, inflammation and glycolysis in macrophages. Conversely, myeloid-specific deletion of HIF-1α impairs macrophage glycolysis and inflammatory polarization. In murine models of obesity, mechanisms of M1 polarization in adipose tissue macrophages are only partly dependent on HIF1 activation. Myeloid-specific deletion of HIF-1α results in decreased inflammatory signaling, decreased CLS formation and an ameliorated metabolic phenotype upon diet-induced obesity (189, 190).

## Epigenetic Control of Macrophage Polarization

Epigenetic mechanisms control chromatin structure and conformation, factors that dictate the accessibility of genetic loci to transcription factors. Epigenetic remodeling, through transcriptional coregulators and epigenetic modifying enzymes (such as histone deacetylases or HDACs), regulates transcription factor activity. Understanding underlying epigenomic regulatory mechanisms can help develop new therapies, for example, by blocking an unwanted pathway or reprogramming macrophages to a more beneficial phenotype.

Rapid induction of an inflammatory transcriptional profile is a hallmark of macrophage activation required for an effective immune response. Under steady state, coregulator complexes bind to genomic regions of a broad repertoire of inflammatory genes to maintain macrophages in a quiescent state, this mechanism avoids deregulated inflammatory gene induction.

**Figure 6:**
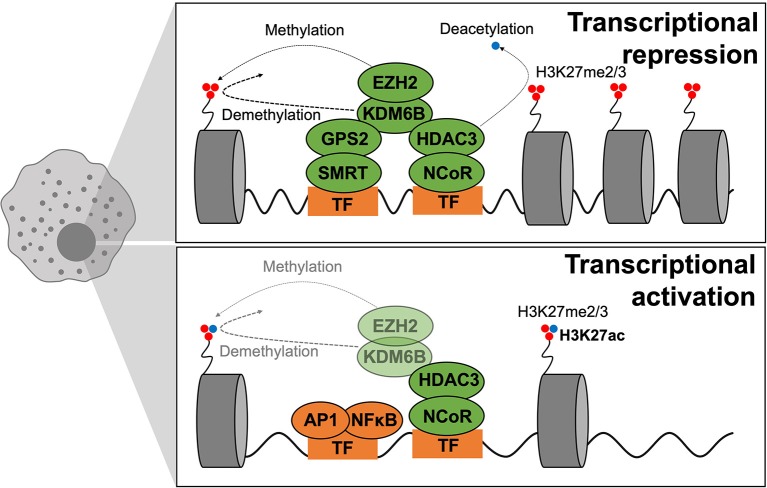
Epigenetics of macrophage polarization in T2D. Epigenetic mechanisms that modulate transcription act on chromatin remodeling and altering DNA accessibility to transcriptional machinery. Modifications are in dynamic exchange where methylation and acetylation status of H3K27 dictate repression or activation of transcription, respectively. When chromatin is closed, methylation is dynamically altered by EZH2 (adding methyl groups) and KDMB6 (removing methyl groups), and the active transcription mark (H3K27ac) is removed through HDAC3 activity. GPS2–SMRT also participate in gene repression. During active transcription, the active transcription mark (H3K27ac) is maintained whilst EZH2–KDM6B play smaller roles. GPS2–SMRT are not present to exert repressive effects.

Coregulators function by first recognizing transcription factor activity and they then modulate this activity by establishing interactions with transcriptional machinery and chromatin (191, 192). Coregulators can be categorized as either coactivators or corepressors. Coactivators recognize and promote active transcription; corepressors however recognize and repress inactive transcription. However, this categorization of coregulator activity does not truly reflect the physiological or physiopathological situation, since coregulator activating or repressive function is highly context dependent. Coregulators establish cell type-dependent and ligand-dependent epigenomes by forming large multiprotein complexes that “write,” “erase,” or “read” reversible chromatin modifications associated with transcriptional activity (Figure 6). Although underlying mechanisms remain to be elucidated, convincing evidence places altered function or expression of coregulators at the center of dysregulated transcription inherent to disease-specific epigenomes.

Two such corepressors are the Nuclear Receptor Corepressor (NCoR1) and silencing mediator of retinoic acid and thyroid hormone receptor (SMRT or NCoR2) that interact with inflammatory transcription factors such as AP-1 and NFκB and in-turn bind to specific *genomic* regions to regulate transcription (192, 193). The classical view was that upon TLR4 stimulation, the NCoR complex is released from promoter/enhancer regions of inflammatory genes to promote, or de-repress, their transcription (194). However, in many cases this distinction does not truly reflect the *in vivo* situation, with context-specific microenvironmental cues dictating coregulator properties. In the context of macrophage polarization and T2D, the specific deletion of NCoR in macrophages caused the transcriptional activation of LXR, leading to the induction of lipogenic genes, which in-turn causes local anti-inflammatory effects by repressing NFκB (185). NCoR exerts pro-inflammatory actions in macrophages. Similarly, to NCoR, it was surprising that macrophages from HDAC3-deficient phenotypes were anti-inflammatory in two independent studies (195, 196). These findings are not consistent with earliest studies showing that HDAC3 and NCoR were shown to assemble a repressive complex *via* interaction with the NFκB subunit p50, necessary for the TLR tolerance phenomena where sustained TLR4 activation represses inflammatory gene expression (192). More mechanistic insights are required to better understand the specific action of NCoR1 and HDAC3.

In contrast, anti-inflammatory functions have been attributed to the SMRT/GPS2 (G-protein pathway suppressor-2) subunit/complex. In our recent study, we demonstrated that macrophage specific knockout of the GPS2 subunit exacerbates metabolic inflammation, aggravating glucose homeostasis under metabolic stress (197). The phenotype is associated with genomic features of the GPS2-repressive pathway, involving direct repression of the c-Jun subunit of AP-1. Considering all the recent studies, 2 sub-complexes may have different functions: GPS2/SMRT may have anti-inflammatory actions whilst NCoR/HDAC3 may act as pro-inflammatory machinery. This could explain the contradictory phenotypes of the respective KO models, despite both being initially classified as corepressor complexes.

Subcomplex specificities would allow controlling transcription of distinct gene clusters in response to a variety of signals and likely result from differential interactions with TFs, coregulators, and chromatin components (e.g., histones). This is exemplified by GPS2 actions in others cell types. In fact, the anti-inflammatory action of GPS2 is conserved in adipocytes by repressing CCL2 and IL6 (198, 199). While the main action of GPS2 in hepatocytes is to repress the metabolic nuclear receptor PPAR-α action (200). Hepatocyte-specific KO of GPS2 is then protective upon inflammatory stimulus while adipocyte- or macrophage-specific GPS2 deficiency is deleterious for whole body glucose homeostasis and exhibits exacerbated inflammation. These opposite functions are also observed in humans (197, 200, 201). These correlations are of importance because they point at the possibility that inappropriate GPS2 function could be linked to macrophage pathways that drive adipose tissue dysfunction and insulin resistance.

Other coregulators, such as Glutamate receptor-interacting protein (GRIP)-1, regulate macrophage programmed responses to IL-4 by acting as a coactivator for Kruppel-like factor (KLF)-4, a known driver of tissue-resident macrophage differentiation (202). Obese mice with conditional macrophage-specific deletion of GRIP1 develop inflammation and substantial macrophage infiltration in metabolic tissues, fatty livers, hyperglycemia and insulin resistance; recapitulating metabolic disease through GRIP-1's glutamate receptor-independent actions. Thus, coregulators such as GPS2, GRIP-1, NCoRs, and HDAC3 are critical regulators of macrophage reprogramming in metabolic disease. Their co-ordinated actions engage transcriptional mechanisms that coordinate the balance between macrophage polarization states and subpopulations to maintain metabolic homeostasis.

Epigenetic remodeling of specific histones is also a mark of macrophage activation states. Macrophage activation can be regulated by trimethylation of lysine residue 27 on histone 3 (a modification annotated as H3K27me3) via the action of lysine-specific demethylase 6B (KDM6B; also known as JMJD3). The histone mark H3K27me3 is represses transcription and is deposited by histone-lysine *N*-methyltransferase (EZH)-2, a subunit of the Polycomb Repressive Complex 2. Whereas, removal of this histone mark is mediated by the H3K27me3 demethylases KDM6A and KDM6B. Zhang et al. reported the critical role of the EZH2 histone methyltransferase modification in altering macrophage phenotype (203). EZH2 controls H3K27me3 deposition on the promoter of SOCS3, that encodes a cytokine signaling repressor. Accordingly, mice with a myeloid-specific deficiency of EZH2 exhibit attenuated macrophage activation and reduced inflammation under models of autoimmune disease. These findings make EZH2 an attractive target for other inflammatory diseases such as T2D.

The role of KDM6B in macrophage polarization is unclear. Pioneer studies have proposed that KDM6B is not necessary for the polarization of the pro-inflammatory macrophage phenotype in mice but is required for a proper anti-inflammatory response via the removal of H3K27me3 from the IRF4 promoter (204, 205). The absence of KDM6B completely blocks the induction of M2 macrophages in mice challenged with helminths or chitin, indicating that the role of KDM6B must be greater in M2 than in M1 macrophages (206). In contrast, Pro-inflammatory TLR4 gene activation was decreased in KDM6B-deficient macrophages. In line with these results, targeting KDM6B H3K27me3 demethylases with small-molecule inhibitors impairs inflammatory responses in human primary macrophages and could thus be of high pharmacological interest for the treatment of inflammatory diseases including T2D (207, 208). Interestingly, KDM6B also modulates expression of chemokines dependent of GM-CSF stimulation, which normally acts via STAT5-mediated and IRF5-mediated induction of a pro-inflammatory phenotype. Epigenetic signatures differ in disease states of chronic inflammation, such as T2D. KDM6B is one of the few epigenetic modifiers that could be directly involved in altering the epigenetic signature of macrophages. Gallagher et al. were the first to report on the role of KDM6B in controlling macrophage expression of IL-12 in a diabetic context (208). Proof-of-principle of these findings was achieved in a recent study where macrophages treated with a selective KDM6B inhibitor showed altered expression of pro-inflammatory cytokines (209). What remains to be determined are the degrees of contribution of nutrient overconsumption and obesity, insulin resistance, or hyperglycaemia to observed changes in histone methylation. A recent study proposes that altered DNA methylation is predominantly a consequence of adiposity, rather than a cause (210).

## Conclusions: Toward Functional Classification, Bioenergetics and Non-Immune Signaling

Important advances have been made in the past decades characterizing the role of tissue macrophages in the development of insulin resistance. Indeed, macrophages are now seen as central actors in maintaining tissue and organism homeostasis in response to daily challenges of transient over- and under- nutrition; from inflammatory signaling necessary for insulin secretion, to the housekeeping roles they play in buffering AT lipolysis and their non-inflammatory signaling in NAFLD.

To date studies have largely focused on deciphering the molecular mechanisms that control macrophage responses to dysmetabolism, with a relatively restrictive categorization into M1-like vs. M2-like macrophages. Recent technological advances of single cell sequencing have allowed a much more in-depth characterization of tissue macrophage subsets that do not neatly adhere to the previously proposed dichotomies. Indeed in other fields of study, namely immune cell ontogeny, single cell sequencing has led to a thorough functional reclassification of innate immune subtypes (16). Such studies have particular value in characterizing macrophages in tissue niches that have been overlooked until recently, like pancreatic islet macrophages or sympathetic nervous system associated macrophages.

Such a shifting paradigm in macrophage functional classification can also be extended to their metabolic characterization, their bioenergetic requirements and adaptations to the specific challenges of insulin resistance. Numerous studies in infection and immunity have largely embraced bioenergetic adaptation as *bona fide* immune cell activation. Tissue macrophage bioenergetics remains to be elucidated, at the developmental stage, at steady state and at the onset of insulin resistance. Macrophage metabolism represents an attractive therapeutic target that will modulate inflammation without drastically altering effector functions by turning the immune response “on” or “off.”

Following recent discoveries of non-immune and non-inflammatory signaling from macrophages, the scientific community has gained insight into non-canonical roles of the innate immune system. Further investigation into such homeostatic non-inflammatory signaling must be carried out in macrophages as well as related innate immune cells, such as dendritic cells, NK cells, and ILCs. As innate immunity, in all its diversity, is known to maintain homeostasis without necessarily engaging inflammation, steady state characterization, and responses to physiological variation must be mapped to gain more basic insight into the deregulation of innate immune effector function that leads to metabolic pathology.

Despite consistently strong associations and mechanistic links between inflammation and insulin resistance there have been relatively few successful translational advances. Current anti-diabetic treatments aim to normalize glycaemia through various mechanisms and have been shown to also buffer systemic inflammation (e.g., TZDs, DPP-4 inhibitors, GLP-1 RAs). Such positive effects attribute improvement in the inflammatory profile to improved metabolic responses (211). Considering the overwhelming evidence that macrophage polarization is central to T2D pathology seemingly few clinical trials target inflammation in T2D.

To date anti-inflammatory strategies in clinical trials have targeted cytokines with neutralizing antibodies (e.g., anti-TNF, anti-IL1) or have applied agents with uncharacterised mechanisms (e.g., chloroquine, diacerein). Studies on these drugs have been promising, improving insulin sensitivity, secretion, or fasting blood glucose (212–214). The main obstacles to their routine application are the lack of long-term studies to evaluate efficacy and safety. Other hurdles to the translatability of anti-inflammatory approaches is the fact that inflammation in T2D is multifactorial, and the disease itself predisposes patients to a slew of complex complications and comorbidities (in which case the rise of precision medicine aims to identify mechanisms of response or those at-risk). Technical barriers also affect translational potential, for example clinical trials evaluate inflammation based on relatively non-specific circulating markers, such as CRP, which at best reflect systemic inflammation. Whereas, in preclinical studies scientists tend to evaluate tissue-specific inflammation, the extrapolation of which to human studies represents a substantial technical hurdle. Specific drug delivery to macrophages also represents a technical challenge and bypassing the cell-specificity leaves the door open to unexpected or unwanted side-effects. In light of the above work, promising approaches are slowly but surely increasing the translational potential of targeting inflammation in metabolic disease, for example the repurposing of well-tolerated drugs from other pathologies or fields, as was the case with anti-malarial chloroquine and hydroxychloroquine, and diacerein used to treat arthritis. In basic research, increasing attention is being placed earlier in disease course, where mechanisms that may delay or negate the natural course of T2D are being described and will soon provide bases for novel therapeutic targets. The development of small-molecule inhibitors or anti-sense oligonucleotides are increasingly attractive when targeting epigenetic or transcriptional pathways and are proving of increasing value to the clinical research community. Similarly, the search for metabolic immunogens or characterization of circulating immune cell populations will allow the development of predictive biomarkers of susceptibility to disease or risk-proxies of disease progression once insulin resistance has been established.

## Author Contributions

LO, ED, KD, NV, and FA wrote the review.

### Conflict of Interest

The authors declare that the research was conducted in the absence of any commercial or financial relationships that could be construed as a potential conflict of interest. The reviewer A-FB declared a shared affiliation, with no collaboration, with all of the authors, to the handling editor at the time of the review.

## Abbreviations

2-DG: 2-deoxyglucose
ABCA/G1: ATP-binding cassette transporter
ACLY: ATP citrate lyase
AMPK: AMP-activated protein kinase
AP-1: Activator protein 1
APOA1: Apolipoprotein A1
ARG1: Arginase 1
AT: Adipose tissue
ATM: Adipose tissue macrophage
ATP: Adenosine triphosphate
CARKL: carbohydrate kinase like (sedoheptulose kinase)
CCL2: Chemokine (C-C motif) ligand 2/monocyte chemoattractant protein 1
CCR2: C-C chemokine receptor 2
CD: Cluster of differentiation
Clec4F: C-type lectin domain family 4 member F
CLS: Crown-like structure
COX2: Cyclooxygenase 2
DAMP: Damage-associated molecular pattern
ETC: Electron transfer chain
EZH2: Enhancer of zeste homolog 2
FA: Fatty acid
FAS: Fatty acid synthase
FATP: Fatty acid transporter protein
FOXO: Forkhead box protein O
GC: Glucocorticoids
GLUT1: Glucose transporter 1
GPS2: G Protein Pathway Suppressor 2
GR: Glucocorticoid receptor
GRIP1: Glutamate receptor interacting protein 1
HDAC: Histone deacetylase
HFD: High-fat diet
HIF1: Hypoxia inducible factor 1
HK: Hexokinase
HRE: Hypoxia response element
HSC: Hepatic stellate cell
IFN: Interferon
IGFBP-7: Insulin-like growth factor binding protein 7
IKK: IκB Kinase
IL: Interleukin
iNOS: inducible nitrous oxide synthase
IRAK: Interleukin-1 receptor associated kinase
IRF: Interferon regulatory factor
IRS: Insulin receptor substrate
JAK: Janus Kinase
JNK: c-Jun N-terminal kinase
KC: Kupffer cell
KDM6B: Lysine demethylase 6B
KLF4: Kruppel-like factor 4
LPL: Lipoprotein lipase
LPS: Lipopolysaccharides
LXR: Liver-X-receptor
Ly6c/g: Lymphocyte antigen 6 c/g
MAPK: Mitogen activated protein kinase
miRNA/miR: Micro RNA
Mme: metabolically activated macrophage
MSR: Macrophage scavenger receptor
MyD88: Myeloid differentiation primary response 88
NADH: Nicotinamide adenine dinucleotide + hydrogen
NADPH: Nicotinamide adenine dinucleotide phosphate
NAFLD: Non-alcoholic fatty liver disease
NASH: Non-alcoholic steatohepatitis
NCoR: Nuclear receptor corepressor
NFκB: Nuclear factor-kB
NLRP3, NACHT: LRR and PYD domains-containing protein 3
NO: Nitrous oxide
NR: Nuclear receptor
OPN: Osteopontin
OXPHOS: Oxidative phosphorylation
PAMP: Pathogen associated molecular pattern
PD1: programmed cell death protein 1
PDGF-R: Platelet-derived growth factor receptor
PDH: Pyruvate dehydrogenase
PFK/uPFK: Phosphofructokinase/ubiquitous Phosphofructokinase
PGC-1a: Peroxisome proliferator-activated receptor gamma coactivator 1-alpha
PKM2: Pyruvate kinase isozyme M2
PPAR: Peroxisome proliferator-activated receptor
PPP: Pentose phosphate pathway
PRR: Pattern recognition receptor
ROS: Reactive oxygen species
SDH: Succinate dehydrogenase
SLC1A5: Solute carrier family 1 member 5
SMRT: Silencing mediator for retinoid or thyroid-hormone receptor
SOCS: Suppressor of cytokine signaling
SREBP: Sterol regulatory element binding protein
STAT: Signal transducer and activator of transcription
T2D: Type-2 diabetes
TCA: Tricarboxylic acid
TGF: Transforming growth factor
Th: T-helper
TIRAP: Till-interleukin 1 receptor domain containing adaptor protein
TLR: Toll-like receptor
TNF: Tumor necrosis factor
TRAM: TRIF-related adaptor molecule
Treg: Regulatory T-cell
TREM2: Triggering receptor expressed on myeloid cells 2
TRIF: Toll-interleukin receptor adaptor inducing interferon-B
UDP-GlcNac: Uridine diphosphate N-acetylglucosamine
VASP: Vasodilator-stimulated phosphoprotein
VLDL: Very-low density lipoproteins.
